# NORAD accelerates chemo-resistance of non-small-cell lung cancer via targeting at miR-129-1-3p/SOX4 axis

**DOI:** 10.1042/BSR20193489

**Published:** 2020-01-24

**Authors:** Qiang Huang, Shijiang Xing, Aiping Peng, Zhiwu Yu

**Affiliations:** 1 Department of Pharmacy, Affiliated Xiaolan Hospital of Southern Medical University, Zhongshan, China; 2 Department of Oncology, Affiliated Xiaolan Hospital of Southern Medical University, Zhongshan, China; 3 Affiliated Xiaolan Hospital of Southern Medical University, Zhongshan, China; 4 Division of Laboratory Science, Affiliated Cancer Hospital and Institute of Guangzhou Medical University, Guangzhou, China

**Keywords:** chemo-resistance, miR-129-1-3p, non-small-cell lung cancer, NORAD, SOX4

## Abstract

Substantial researches indicated that long non-coding RNAs (lncRNAs) exerted profound effects on chemo-resistance in cancer treatment. Nonetheless, the role of NORAD in non-small-cell lung cancer (NSCLC) remains unclear. In the present study, we chose NSCLC cell lines H446 and A549 to explore the function of non-coding RNA activated damage (NORAD) in response to cisplatin (DDP) resistance of NSCLC. Experimental data manifested that NORAD was up-regulated in DDP-resistant NSCLC tissues and cells. NSCLC patients with high NORAD expression suffered a poor prognosis. NORAD knockdown resensitized H446/DDP and A549/DDP to DDP. Besides, NORAD acted as a molecular sponge of miR-129-1-3p. MiR-129-1-3p showed a low level of expression in DDP-resistant NSCLC tissues. Moreover, miR-129-1-3p overexpression impaired DDP resistance in H446/DDP and A549/DDP cells. SOX4 was the downstream target of miR-129-1-3p. Especially, SOX4 overexpression offset the effects of NORAD silence on H446/DDP and A549/DDP cells resistance to DDP. NORAD knockdown resensitized H446/DDP and A549/DDP to DDP in NSCLC via targeting miR-129-1-3p/SOX4 axis, offering a brand-new target for NSCLC chemo-resistance.

## Introduction

Lung cancer is considered as one of the most malignant cancers with high death rates around the world. Non-small cell lung cancer (NSCLC) counts for around 85% of all cases [1]. Based on platinum, especially cisplatin (DDP), chemotherapy is a main treatment strategy for NSCLC after surgical resection [2]. Unfortunately, cisplatin resistance influences the desirable effects of DDP in clinical therapy, consequently lead to high death rates of NSCLC. [3].Thus, it is crucial to gain an insight into DDP resistance in NSCLC to overcome DDP resistance and improve the unsatisfactory outcomes results of cisplatin treatment for NSCLC.

Despite over 200 nucleotides in length, long non-coding RNAs (lncRNAs) are a family of RNAs without potential to be translated into proteins [4]. It has been found to be involved in substantial biological activities of cancers including metastasis [5], angiogenesis [6], drug resistance [7] and autophagy [8]. Non-coding RNA Activated Damage (NORAD) is a lncRNA induced by DNA damage. Lately, it was found to play essential role in for stabilizing gene expression [9]. A previous study indicated that NORAD overexpression was associated with accelerating progression of colorectal cancer [10]. Moreover, it was discovered as a tumor promoter in colorectal cancer [11]. But its detailed function in DDP resistance has not been elucidated in NSCLC cells.

To date emerging studies have supported the novel regulatory system termed competing endogenous RNAs (ceRNAs) network, where lncRNAs acted as miRNAs sponges to moderate the expression of miRNAs so as to release their downstream targets from miRNA-induced silencing complex [12–14]. UCA1 acted as an oncogene in NSCLC through sponging miR-193a-3p [15]. In our study, we analyzed that NORAD functioned as a ceRNA to regulate the process of NSCLC resistance to DDP.

Our study mainly intended to investigate the role of NORAD in moderating the course of NSCLC resistance to DDP. NORAD was identified to enhance chemo-resistance in NSCLC through sponging miR-129-1-3p to elevate SOX4 expression, suggesting that knockdown of NORAD would be conducive to overcome DDP resistance in NSCLC chemo-therapy treatment.

## Materials and methods

### Tissue samples

Sixty paired NSCLC tissues and adjacent normal tissues were collected from patients in Affiliated Xiaolan Hospital of Southern Medical University between July 2013 and August 2018. Tissue samples were gathered from patients with NSCLC who were responded to cisplatin (DDP-sensitive) (*n* = 32), patients with NSCLC who were unresponsive to cisplatin (DDP-resistant) (*n* = 28), and normal lung tissue from healthy individuals (*n* = 60). All patients underwent surgery resection at Affiliated Xiaolan Hospital of Southern Medical University. No patients underwent radiotherapy or immunotherapy before surgery. Fresh tissues were frozen in liquid nitrogen and preserved at −80°C. All patients provided written informed consent before the study, which was approved by the Ethics Committee of Affiliated Xiaolan Hospital of Southern Medical University.

### Cell culture and treatment

Normal human lung epithelium cell (BEAS-2B) and NSCLC cells (H446, A549) were bought from American Type Culture Collection (Manassas, VA, U.S.A.). Cells were cultured at 37°C in an incubator containing 5% CO_2_ in DMEM (Invitrogen, Carlsbad, CA, U.S.A.) supplied with 10% FBS (Invitrogen), 1% penicillin/streptomycin (Sigma-Aldrich, Milan, Italy). Cisplatin (DDP; Sigma-Aldrich) was used for treating H446 and A549 cell lines for drug-resistance study as previously illustrated [16].

### Cell transfection

H446 and A549 cells were transfected with specific shRNAs against NORAD (sh-NORAD#1#2) and the negative control (shNC), pcDNA3.1/NORAD, pcDNA3.1/SOX4 and their empty pcDNA3.1 vector (GenePharma, Shanghai, China), respectively. The miR-129-1-3p mimics, miR-129-1-3p inhibitor, NC mimics and NC inhibitor were constructed by GenePharma. Each plasmid was transfected into cells using Lipofectamine 2000 (Invitrogen).

### RT-qPCR

Total RNA from cells was extracted by TRIzol Reagent (Invitrogen). RNAs were reverse transcribed into cDNA with utilization of a reverse transcription kit (Takara, Dalian, China). RT-qPCR was performed using the SYBR Green PCR Kit, which was purchased from Takara. Fold expression changes were calculated with 2^−ΔΔCt^ method, and GAPDH/U6 served as internal controls.

### MTT assay

A total of 1 × 10^3^ cells were inoculated in 96-well plates with various concentrations of DDP over specific time points. The cell vitality of NSCLC cells was exposed with different concentration (0, 0.25, 0.5, 1, 2, 4, 8, 16 and 32 μg/ml) of DDP. About 2 μg/ml DDP was treated in H446 and A549 cells to study the viability variation with their parental ones. About 20 µl MTT reagents were added for incubating for additional 4 h. Subsequently, dimethyl sulfoxide (DMSO) was added to dissolve the crystals. The determination of optical density at 490 nm was measured by using a microplate reader (Bio-Tek Instruments, Hopkinton, MA, U.S.A.).

### Colony formation assay

Cells were incubated in 6-well plates with the density of 500 cells/well and then incubated for 2 weeks. Then, Crystal Violet (Solarbio, Beijing, China) was applied for dying colonies after being fixed in paraformaldehyde (Sigma-Aldrich). Visible colonies were counted via microscopy (Nikon, Tokyo, Japan).

### Western blot

Proteins were extracted using RIPA buffer and qualified through BCA kit (Solarbio). The proteins were separated by SDS-PAGE and then moved to PVDF membranes. After being blocked with skimmed milk, target proteins were incubated with primary antibodies: anti-SOX4 (ab236557, Abcam, Cambridge, U.S.A.) and anti-GAPDH (ab8245). Secondary antibodies were added for 1 h. Last, proteins were examined by chemiluminescence detection system.

### Apoptosis assay

H446 and A549 cells transfected with sh-NORAD#1/2 or sh-NC were incubated in 6-well plates for cultivation. Cells were re-suspended in 1× binding buffer containing FITC‐annexin V and propidium iodide (PI) for double staining after being fixed in 70% ice-cooled ethanol (Sigma-Aldrich) for 2 h. Finally, apoptosis rate were determined by Flow cytometry (BD Biosciences, Franklin Lakes, NJ, U.S.A.).

### RNA pull-down assay

Cell lysates were separately incubated with biotinylated RNA including Bio-NORAD-WT, Bio-NORAD-Mut, Bio-miR-129-1-3p-WT, Bio-miR-129-1-3p-Mut and Bio-NC overnight. Afterward, M-280 streptavidin magnetic beads (Sigma-Aldrich) were added for co-culturing for 48 h. At last, relative RNA expression level in pulled-down complex was evaluated by RT-qPCR.

### TUNEL assay

The apoptosis of H446 and A549 cells were assessed via TUNEL Apoptosis Kit (Invitrogen). The above cells were dyed by the use of DAPI (Koritai Biotechnology, Beijing, China). Then, cells were captured by fluorescence microscopy (Olympus, Tokyo, Japan).

### Luciferase reporter assay

The wild-type (WT) and mutant (Mut) binding sites of miR-129-1-3p in NORAD sequence or SOX4 3′UTR were sub-cloned into pmirGLO dual-luciferase vector to construct luciferase reporter vectors, named as NORAD-WT/Mut and SOX4-WT/Mut. And then vectors were transfected severally with miR-129-1-3p mimics or NC mimics into H446 and A549 cells. The luciferase activity was examined after 48 h by Dual-Luciferase Reporter Assay System (Promega, MA, U.S.A.).

### RIP assay

Magna RNA-Binding Protein Immunoprecipitation Kit (Millipore, MA, U.S.A.) was employed for conducting RIP assay. Briefly, cell extract was incubated with RIP buffer containing magnetic beads conjugated with human anti-Ago2 antibodies (Millipore) or anti-IgG (Millipore). The RT-qPCR was performed for determining the relative enrichment of NORAD, miR-129-1-3p and SOX4 in the precipitates.

### Statistical analysis

SPSS (SPSS Inc., Chicago, IL, U.S.A.) was applied for the statistical analysis and data were showed as mean ± SD. One-way ANOVA analysis or Student’s *t*-test was employed for the difference between groups, with significant level of *P* < 0.05. Overall survival curve was evaluated through Kaplan–Meier method. Each experimental procedure was repeated for more than two times.

## Results

### NORAD was overexpressed in NSCLC tissues and cells

**Figure 1:**
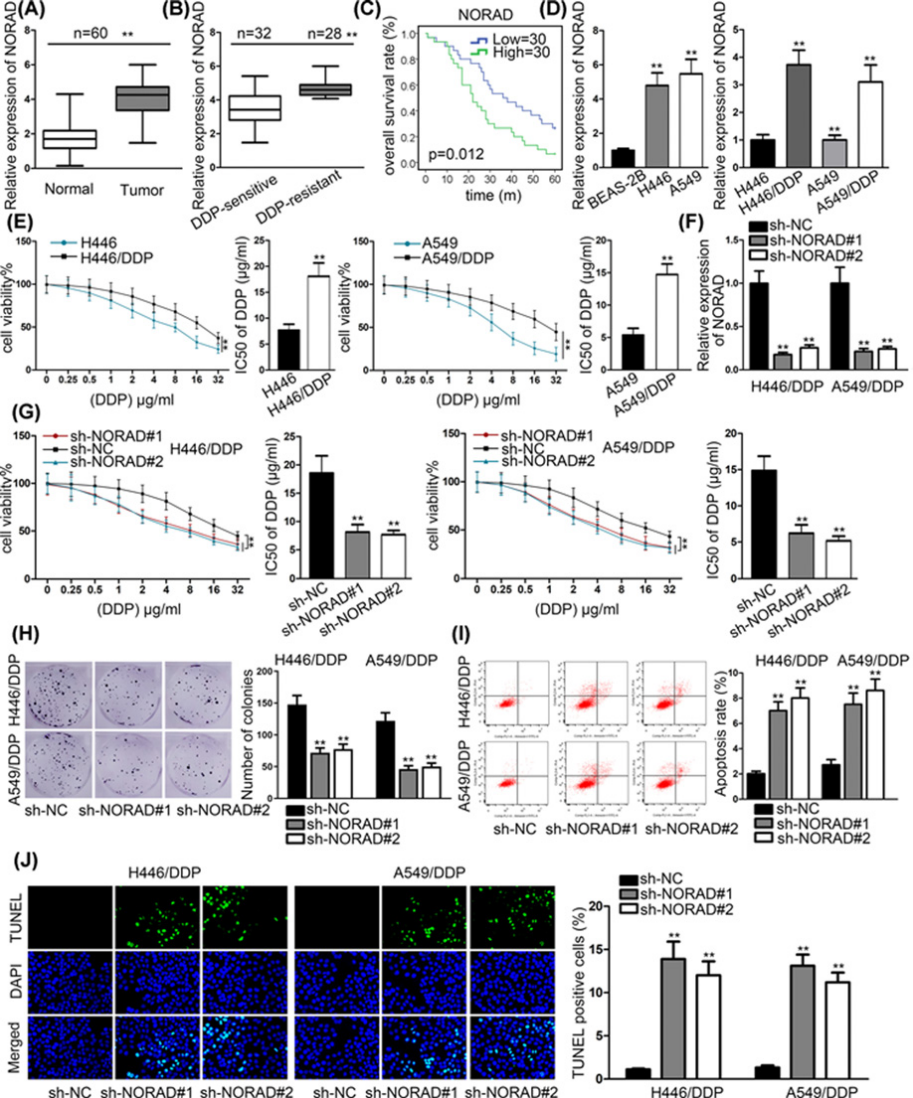
NORAD expression was lifted in NSCLC tissues and cells (**A**) RT-qPCR assays were used to test the expression of NORAD in NSCLC tissues and adjacent normal tissues. (**B**) NORAD expression was examined in DDP-sensitive and DDP-resistant tissues. (**C**) Kaplan–Meier curve was used to analyze the overall survival between high and low NORAD expression. (**D**) N0RAD expression was tested in NSCLC cell lines (H446 and A549) and normal human lung bronchial epithelial BEAS-2B cell as well as their DDP-resistant cell H446/DDP and A549/DDP cells. (**E**) MMT was conducted to evaluate cell viability of H446 and A549 while their parental cells were treated with DDP. (**F**) RT-qPCR assays were conducted to appraise the efficiency of sh-NORAD#1/2 in cells. (**G** and **H**) MTT and colony formation assays were conducted to examine the cell proliferation while H446/DDP and A549/DDP transfected with sh-NORAD#1/2 exposed to DDP. (**I** and **J**) Flow cytometry analysis and TUNEL were conducted to probe rate of apoptosis in H446/DDP and A549/DDP transfected with sh-NORAD#1/2 after exposure of 2 μg/ml DDP; **P < 0.01.

To explore the role of NORAD in NSCLC, we tested the expression of NORAD in 60 pairs NSCLC tissues and adjacent normal tissues by RT-qPCR assays. In contrast with adjacent normal tissues, NORAD expression was notably higher in NSCLC tissues (Figure 1A). Subsequently, NORAD expression was evidently higher in DDP-resistant NSCLC tissues compared with DDP-sensitive NSCLC tissues (Figure 1B). Kaplan–Meier survival analysis manifested that NSCLC patients with high NORAD expression had poor prognosis (Figure 1C). Next, we chose H446 and A549 cell from NSCLC cell lines to detect NORAD expression. RT-qPCR results revealed that NORAD was overexpressed in H446 and A549 cell compared with normal human lung bronchial epithelial BEAS-2B cell. Moreover, H446/DDP and A549/DDP cells revealed significant higher NORAD expression than their parental cells (Figure 1D). To appraise the resistance of H446/DDP and A549/DDP cells to DDP, we carried out MTT assay to test IC50 of DDP in H446/DDP and A549/DDP cells and their parental H446 and A549 cells. The increased IC50 proved that H446/DDP and A549/DDP cells were resistant to DDP compared with H446 and A549 cells (Figure 1E). Then, sh-NORAD#1 and sh-NORAD#2 were transfected into H446/DDP and A549/DDP cells to knockdown NORAD expression. The results of RT-qPCR assays showcased that NORAD expression declined by sh-NORAD#1/2 remarkably (Figure 1F). To further explore NORAD effects on the resistance of H446/DDP and A549/DDP to DDP, MTT and colony formation assays were conducted. The outcomes exhibited that knockdown of NORAD dropped the cell viability and inhibited the proliferation in H446/DDP and A549/DDP cells (Figure 1G,H). However, previous study has experimentally revealed that NORAD silencing dampen NSCLC cell proliferation [17]. It was probable that the negative impact on cell viability/proliferation was due to the interfering role of sh-NORAD on cell proliferation. Therefore, we performed MTT assay in H449 and A549 cells (with or without DDP treatment). Its results showed that the cell viability was suppressed by silencing NORAD in H449/DDP and A549/DDP cell lines, as well as their parental ones. Notably, compared with the parental H449 and A549, the cell viability was hampered by sh-NORAD in a more distinct manner in H449/DDP and A549/DDP (Supplementary Figure S1A), indicating that NORAD knockdown suppressed cell viability via resensitizing H446/DDP and A549/DDP to DDP. To further verify this assumption, we constructed colony formation assay to detect the influence of NORAD knockdown on cell proliferation in H449 and A549 cells (with or without DDP treatment). Its results further validated that NORAD silencing hampered proliferation via resensitizing H446/DDP and A549/DDP to DDP (Supplementary Figure S1B). To investigate the influence of NORAD on DDP-mediated apoptosis, we performed flow cytometry analysis and TUNEL assays in H446/DDP and A549/DDP with 2 μg/ml DDP. The consequence manifested that NORAD silencing conspicuously enhanced DDP-induced apoptosis in H446/DDP and A549/DDP cells (Figure 1I,J). To summarize, NORAD was up-regulated in NSCLC tissues and cells. Knockdown of NORAD resensitized H446/DDP and A549/DDP to DDP.

### NORAD acted as a miR-129-1-3p sponge in NSCLC

**Figure 2:**
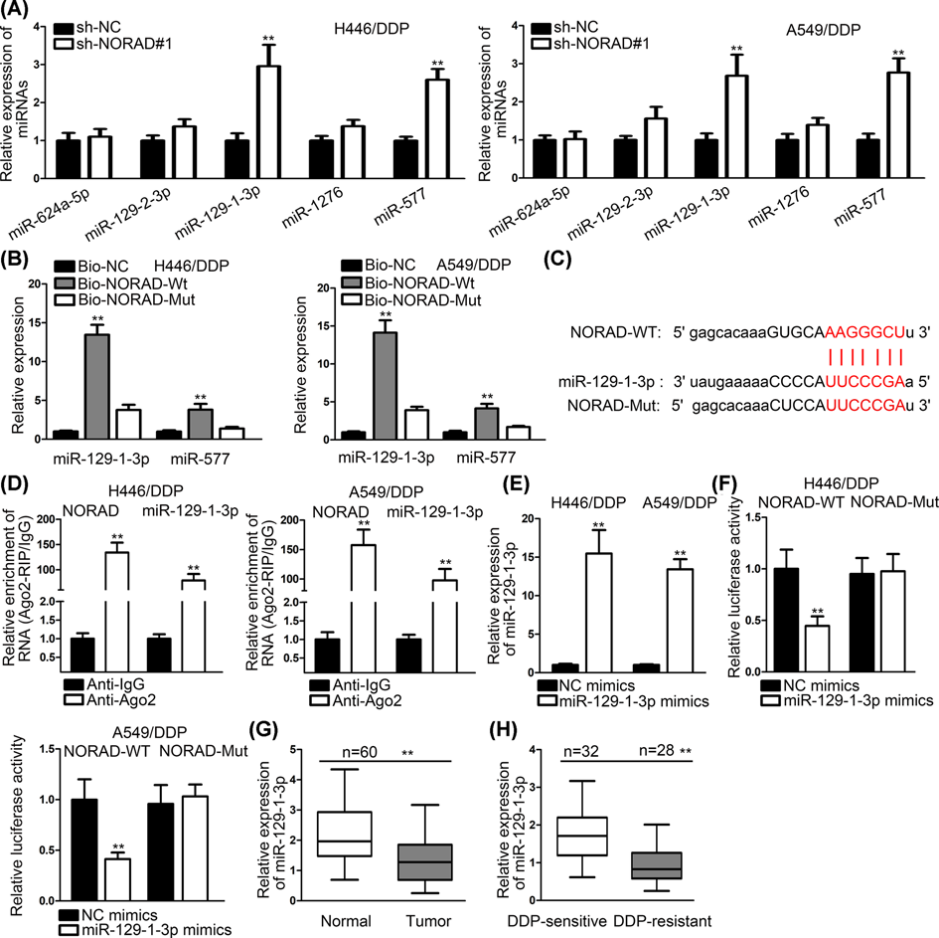
NORAD acted as a miR-129-1-3p sponge in NSCLC (**A**) The expression of miRNAs was appraised in cells transfected with sh-NORAD#1. (**B**) RNA pull-down assays were applied to see the combination between miRNAs and NORAD. (**C**) The binding sites between miR-129-1-3P and NORAD were shown by bioinformatics. (**D**) RIP assays were conducted to illustrate miR-129-1-3p and NORAD coexisted in RISCs. (**E**) The expression of miR-129-1-3p was examined in cells transfected with miR-129-1-3p mimics. (**F**) Luciferase reporter assays were conducted to prove the combination between miR-129-1-3p and NORAD. (**G**) MiR-129-1-3p expression was tested in NSCLC tissues and adjacent normal tissues. (**H**) MiR-129-1-3p expression was evaluated in DDP-sensitive and DDP-resistant tissues; **P < 0.01.

To further analyze the potential mechanism of NORAD-mediated H446/DDP and A549/DDP resistance to DDP, we searched starBase to explore its downstream combinable miRNAs. We found that five miRNAs could bind to NORAD in our set condition. We transfected sh-NORAD#1 into cells and estimated the expression of these miRNAs. The results depicted that expressions of miR-129-1-3p and miR-577 were obviously higher than other miRNAs (Figure 2A). Then, we performed RNA pull-down assays. The outcomes showed that biotinylated miR-129-1-3p could pull down NORAD more than biotinylated miR-577 (Figure 2B). Binding sites between miR-129-1-3p and NORAD were shown in Figure 2C. RIP assays were conducted to evaluate the mutual interaction. We observed that both NORAD and miR-129-1-3p were enriched by antibody targeting Ago2 instead of antibody targeting IgG (Figure 2D). This phenomenon indicated miR-129-1-3p and NORAD were coexisted in RNA-induced silencing complexes (RISCs). MiR-129-1-3p mimics were transfected into cells, and the results of RT-qPCR disclosed that miR-129-1-3p expression was increased by miR-129-1-3p mimics (Figure 2E). Then, luciferase reporter assays were conducted. Plasmid containing NORAD-WT possessed significantly low activity after co-transfecting with miR-129-1-3p mimics, but not in that set with NORAD-Mut (Figure 2F). Furthermore, we detected the expression of miR-129-1-3p in 60 pairs NSCLC tissues and adjacent normal tissues. The outcomes revealed that miR-129-1-3p expression was low in NSCLC tissue compared with normal tissue (Figure 2G). More interestingly, we also found that miR-129-1-3p expression was lower in DDP-resistant tissues compared with DDP-sensitive tissues (Figure 2H). In short, all the results demonstrated that NORAD could bind to miR-129-1-3p.

### MiR-129-1-3p overexpression weakened DDP resistance in NSCLC

**Figure 3:**
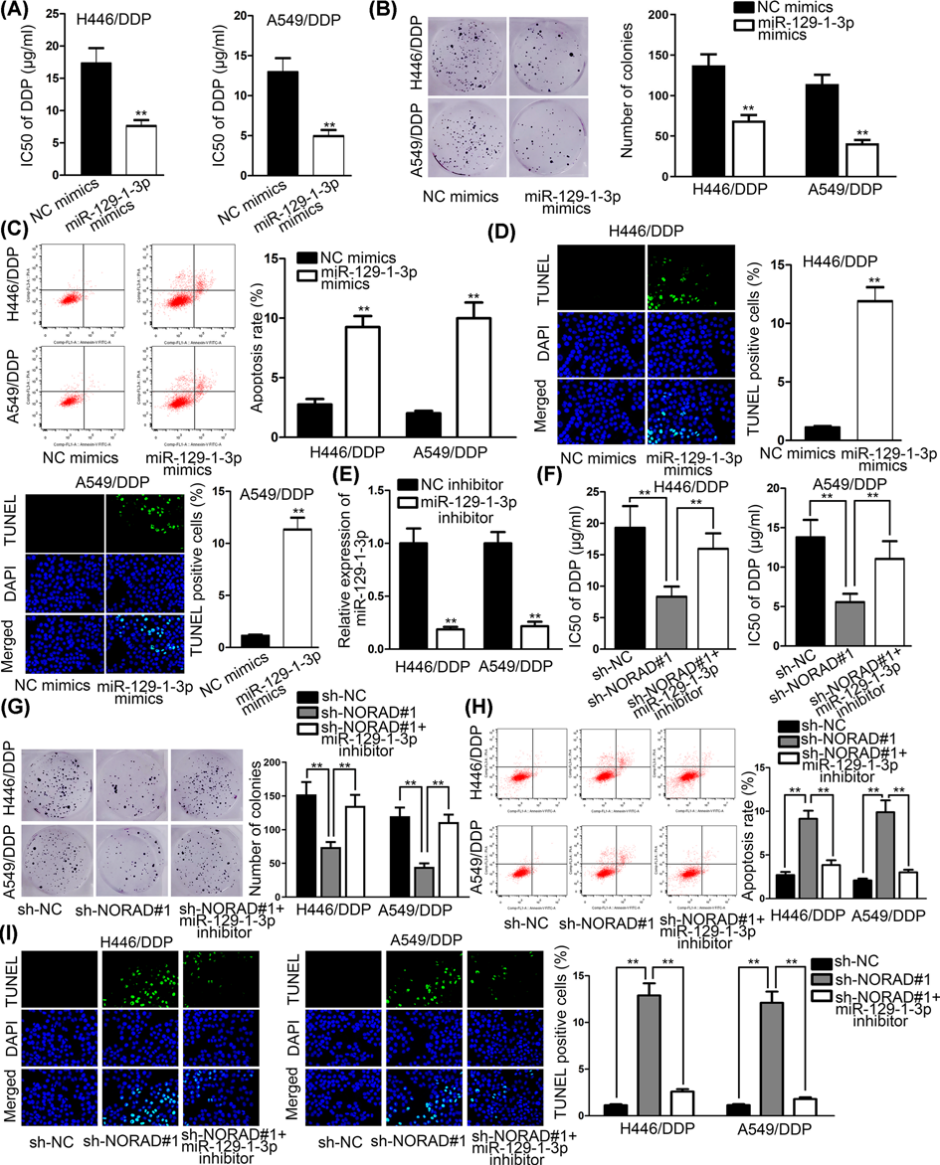
MiR-129-1-3p overexpression weakened DDP resistance in NSCLC (**A** and **B**) MTT revealed the IC50 value in DDP-resistant cells transfected with miR-129-1-3p mimics; and colony formation assays were used to test cell proliferation and H446/DDP and A549/DDP cells treated with miR-129-1-3p mimics;. (**C** and **D**) Flow cytometry analysis and TUNEL were applied to examine apoptosis in cells transfected with miR-129-1-3p mimics and treated with DDP. (**E**) MiR-129-1-3p expression was detected in cells transfected with miR-129-1-3p inhibitor. (**F** and **G**) MTT and colony formation assays were carried out to determine cell proliferation in cells transfected with sh-NORAD#1 and miR-129-1-3p inhibitor and treated with DDP. (**H** and **I**) Flow cytometry analysis and TUNEL were carried out to examine apoptosis rate in cells transfected with sh-NORAD#1 and miR-129-1-3p inhibitor and treated with DDP; **P < 0.01.

Then, we carried out functional assays to study the effects of miR-129-1-3p resistance to DDP in NSCLC. MiR-129-1-3p mimics were transfected into H446/DDP and A549/DDP cells and the results of MTT and colony formation assays depicted that miR-129-1-3p overexpression weakened resistance to DDP in NSCLC (Figure 3A,B). The results of flow cytometry analysis and TUNEL assays manifested that miR-129-1-3p up-regulation elevated apoptosis rate in DDP/mediated NSCLC cells (Figure 3C,D). To further confirm whether NORAD intensified DDP resistance in NSCLC by regulating miR-129-1-3p expression, H446/DDP and A549/DDP cells were transfected with NC inhibitor as normal control and miR-129-1-3p inhibitor into cells. And the results showed that miR-139-1-3p expression was declined by miR-129-1-3p inhibitor (Figure 3E). Outcomes of MTT and colony formation assays suggested that miR-129-1-3p inhibitor reversed the declined DDP resistance in NSCLC cells caused by NORAD knockdown (Figure 3F,G). The influence of NORAD and miR-129-1-3p on DDP-mediated on apoptosis was appraised by flow cytometry analysis and TUNEL. The results disclosed that knockdown of NORAD remarkably enhanced DDP-mediated apoptosis while miR-129-1-3p inhibitor successfully decreased apoptosis rate made by knockdown of NORAD (Figure 3H,I). In brief, miR-129-1-3p overexpression weakened DDP resistance in NSCLC. NORAD silenced overcame DDP resistance in DDP-resistance NSCLC cells by promoting miR-129-1-3p expression.

### SOX4 worked as a functional target of miR-129-1-3p in NSCLC

**Figure 4:**
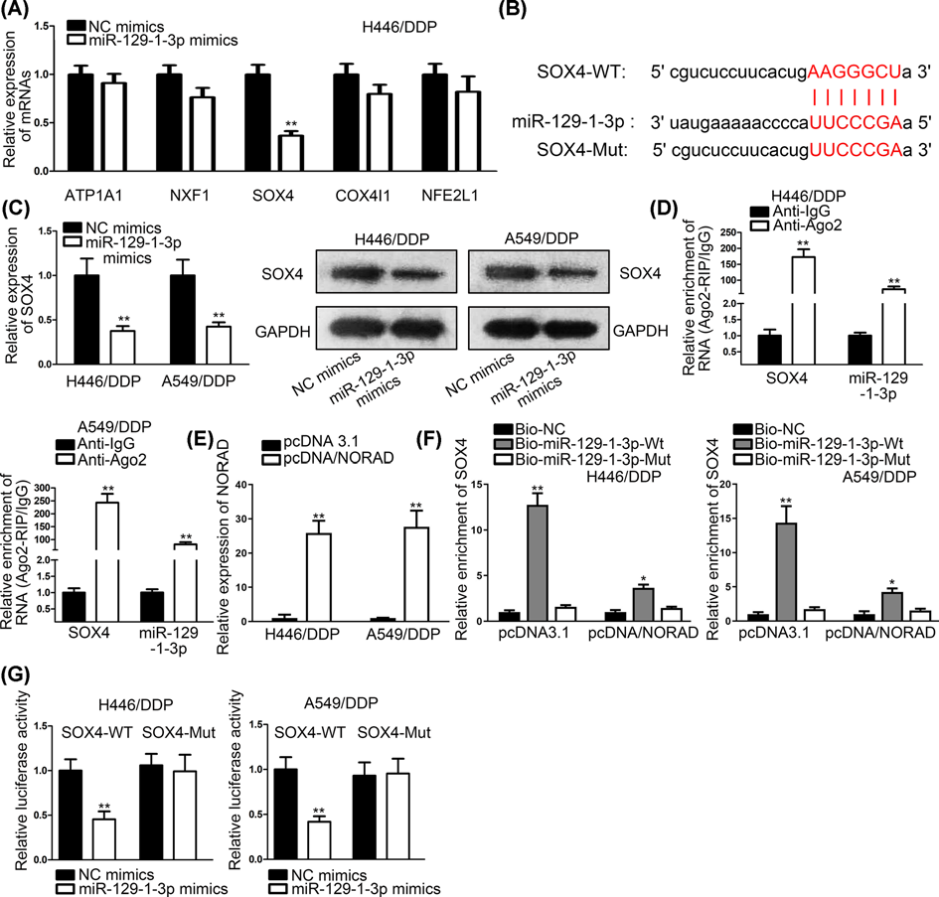
SOX4 worked as a functional target of miR-129-1-3p in NSCLC (**A**) The candidate miRNAs expressions were detected by RT-qPCR assays in cells transfected with miR-129-1-3p mimics. (**B**) Bioinformatics predicted putative binding sites between miR-1291-1-3p and SOX4. (**C**) SOX4 expression and protein were detected in cells transfected with miR-129-1-3p mimics. (**D**) RIP assays were conducted to confirm miR-129-1-3p and SOX4 coexisted in RISCs. (**E**) The overexpressing efficiency of pcDNA3.1/NORAD was evaluated by RT-qPCR assay. (**F**) RNA pull-down assays were conducted to investigate the effects of NORAD overexpression on the interaction between miR-129-1-3p and SOX4 in H446/DDP and A549/DDP cells. (**G**) Luciferase reporter assays were conducted to verify the combination between miR-129-1-3p and SOX4; *P < 0.05, **P < 0.01.

To find out the downstream target of miR-129-1-3p, we searched starBase database and narrow the scope of combinable targets by restricting binding criteria (Clade: mammal; Genome: human; Assembly: hg19; microRNA: miR-129-1-3p; Clip data: high stringency ≥ 3; Degradome data: medium stringency ≥ 2; AgoExpNum: ≥ 5). Five mRNAs like ATP1A1, NXF1, SOX4, COX4I1 and NFE2L1 satisfied our requirements. After transfecting miR-129-1-3p mimics and NC into cells, we found that SOX4 expression declined the most dramatically among them (Figure 4A). Thus, SOX4 was selected out as a target of miR-129-1-3p. Bioinformatics tool predicted the putative binding sites between SOX4 and miR-129-1-3p (Figure 4B). After transfecting miR-129-1-3p mimics into H446/DPP and A549/DPP cells, we used RT-qPCR assays and Western blot to examine the expression of SOX4 mRNA and protein. It was found that SOX4 mRNA and protein were both decreased by miR-129-1-3p mimics (Figure 4C). RIP assays depicted that miR-129-1-3p and SOX4 were coexisted in RISCs (Figure 4D). We forced the overexpression of NORAD by transfection of pcDNA3.1/NORAD into H446/DDP and A549/DDP cells (Figure 4E). RNA pull-down assays indicated that biotinylated miR-129-1-3p-WT could pull down SOX4. However, the biotinylated miR-129-1-3p-WT induced SOX4 enrichment was dramatically decreased in response to overexpressing NORAD (Figure 4F). This phenomenon elucidated that NORAD overexpression weakened the interaction between miR-129-1-3p and SOX4. Then, we applied luciferase reporter assays and the consequences manifested that SOX4-WT-built plasmid’s activity was declined significantly by miR-129-1-3p mimics, while there was no distinct change could be found in SOX4-Mut-set plasmid’s activity (Figure 4G). In a word, SOX4 was a direct target of miR-129-1-3p.

### NORAD knockdown resensitized H446/DDP and A549/DPP cells to DDP via down-regulating g SOX4 expression

**Figure 5:**
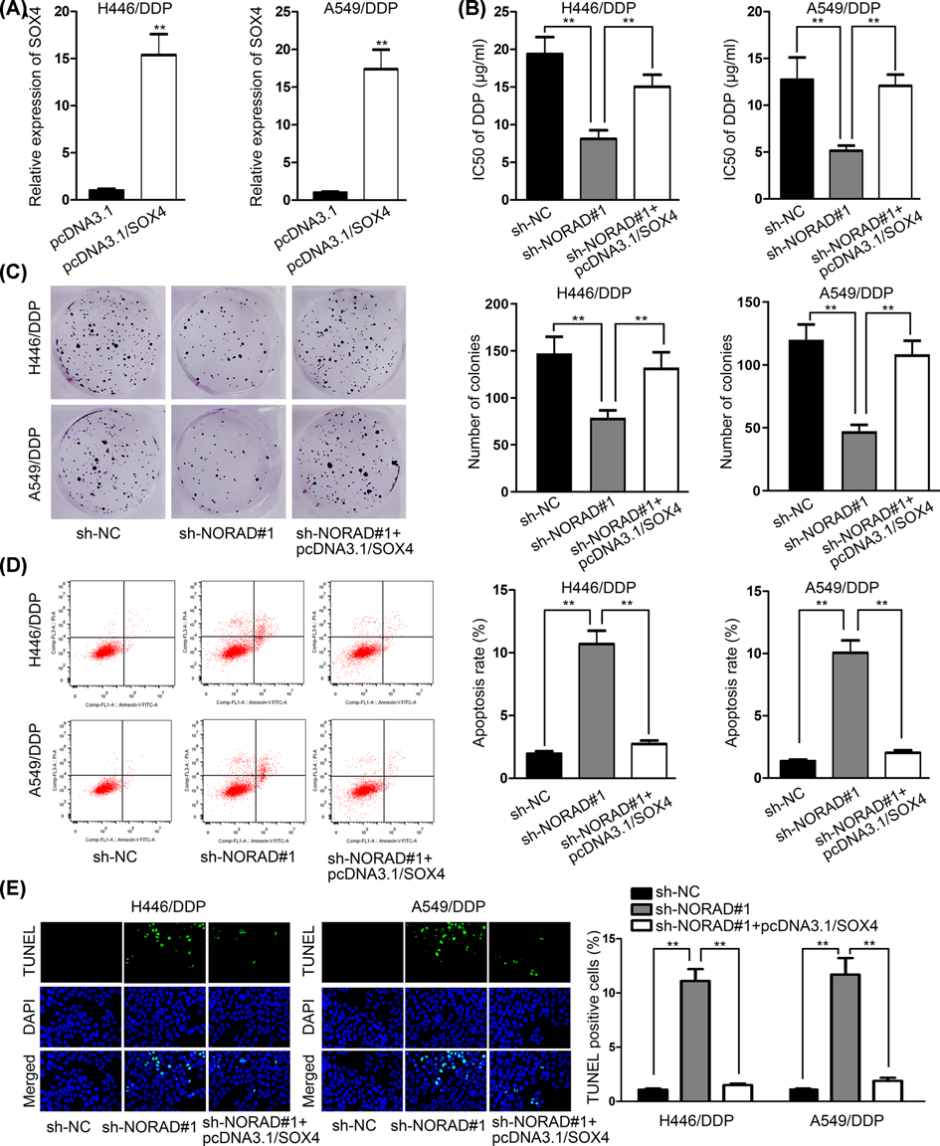
NORAD knockdown enhanced DDP sensitivity of H446/DDP and A549/DPP cells via restricting SOX4 expression (**A**) SOX4 expression was tested in cells transfected with pcDNA3.1/SOX4. (**B** and** C**) MMT and colony formation assays were conducted to measure cell proliferation in cells transfected with sh-NORAD#1 and pcDNA3.1/SOX4 treated with DDP MMT assay revealed the IC50 value for cells under indicted transfections(**D** and **E**) Flow cytometry analysis and TUNEL were carried out to measure apoptosis rate in cells transfected with sh-NORAD#1 and pcDNA3.1/SOX4 treated with DDP; **P < 0.01.

To analyze how NORAD knockdown affected NSCLC cells resistant to DDP, H446/DDP and A549/DDP, cells were transfected with pcDNA3.1 and pcDNA3.1/SOX4. The outcomes of RT-qPCR assays revealed that SOX4 expression was elevated by pcDNA3.1/SOX4 (Figure 5A). The results of MTT assay showed that NORAD silence resensitized H446/DDP and A549/DPP cells to DDP, while pcDNA3.1/SOX4 reversed this effect, as evidenced by the alternation of IC50 (Figure 5B). Colony formation assay showed that the colonies were decreased by silencing NORAD, but increased by SOX4 overexpression (Figure 5C). Flow cytometry analysis manifested that the apoptosis rate was increased by NORAD knockdown, yet dropped by SOX4 overexpression, suggesting that NORAD knockdown promoted DDP-induced apoptosis via regulating SOX4 (Figure 5D). TUNEL assay further confirmed this finding with results showing that overexpression of SOX4 counteracted the influences induced by NORAD knockdown on TUNEL positive cells (Figure 5E). To sum up, NORAD knockdown enhanced NSCLC cells’ DDP sensitivity by down-regulating SOX4 expression.

## Discussion

DDP is usually used in chemotherapy for NSCLC treatment. However, DDP resistance restricted the effectiveness of chemotherapy among NSCLC patients [18]. Therefore, it is critical to elucidate the underlying molecular mechanism of DDP and identify novel target for combating DDP resistance in NSCLC. Mounting studies have indicated that the aberrantly expressed lncRNAs were involved in regulating chemo-resistance in various cancers [8,19]. In the present study, we observed that NORAD was extremely high expressed in DDP-resistance tissues and cells of NSCLC. In addition, NORAD knockdown resensitized DDP sensitivity of NSCLC cells. Mechanically, NORAD knockdown resensitized of NSCLC cells to DDP by acting as a sponge of miR-129-1-3p. Thus, NORAD was verified to exert crucial effects on DDP resistance, suggesting a worthy therapeutic target for DDP resistance in NSCLC.

Substantial researches have uncovered that lncRNAs functioned as ceRNAs to modulate gene expression to affect physiological and pathological course of tumors [20]. NORAD was discovered as an oncogene in bladder cancer [21], osteosarcoma [22] and gastric cancer [23]. In our present study, we focused on the probable ceRNA mechanism of NORAD in mediating DDP resistance in NSCLC cells. NORAD knockdown was revealed to resensitize H446/DDP and A549/DDP cells to DDP by repressing miR-129-1-3p expressions. In addition, we used rescue assays to verify that NORAD knockdown hampered NSCLC cells resistance to DDP via down-regulating miR-129-1-3p-mediated SOX4 expression.

MiRNAs have been reported to be involved in a wide array of cellular behaviors in multiple cancers, such as cell migration [24], proliferation [25] and chemo-resistance [26]. MiR-129-1-3p was reported to function as a tumor inhibitor in gastric cancer [27]. In our study, we found that miR-129-1-3p was down-expressed in NSCLC tissues and DDP-resistant tissue. Moreover, miR-129-1-3p mimics could attenuate DDP-resistant in H466/DDP and A549/DDP cells. However, miR-129-1-3p inhibitor could counteract the ameliorating effect caused by NORAD knockdown on DDP-resistance in NSCLC cells.

A large body of researches have illustrated that SOX4 exert oncogenic function in various cancers, such as squamous cell carcinoma [28], melanoma [29] and breast cancer [30]. In our study, we found that overexpression of SOX4 could counteract the influence caused by knockdown NORAD knockdown on DDP-resistance in NSCLC cells. Consistent with our finding, CCAT1 enhanced NSCLC cells resistance to DDP via targeting SOX4 in NSCLC cells [16].

In summary, the data from our study revealed that NORAD/miR-129-1-3p/SOX4 axis played crucial regulatory role in NSCLC cells to cisplatin resistance, offering underlying targets to weaken DDP resistance in the process of NSCLC.

## Supplementary Material

Supplementary Figure S1

## Abbreviations

lncRNA: long non-coding RNA
NORAD: noncoding RNA activated damage
NSCLC: non-small-cell lung cancer
