# Supplementary material for: NORAD accelerates chemo-resistance of non-small-cell lung cancer via targeting at miR-129-1-3p/SOX4 axis
Source: Biosci Rep. 2020 Jan 24;40(1):BSR20193489. doi: 10.1042/BSR20193489 (PMC6981097; doi:10.1042/BSR20193489)
Supplement: Supplementary Figure S1 [file BSR-2019-3489_supp.pdf]

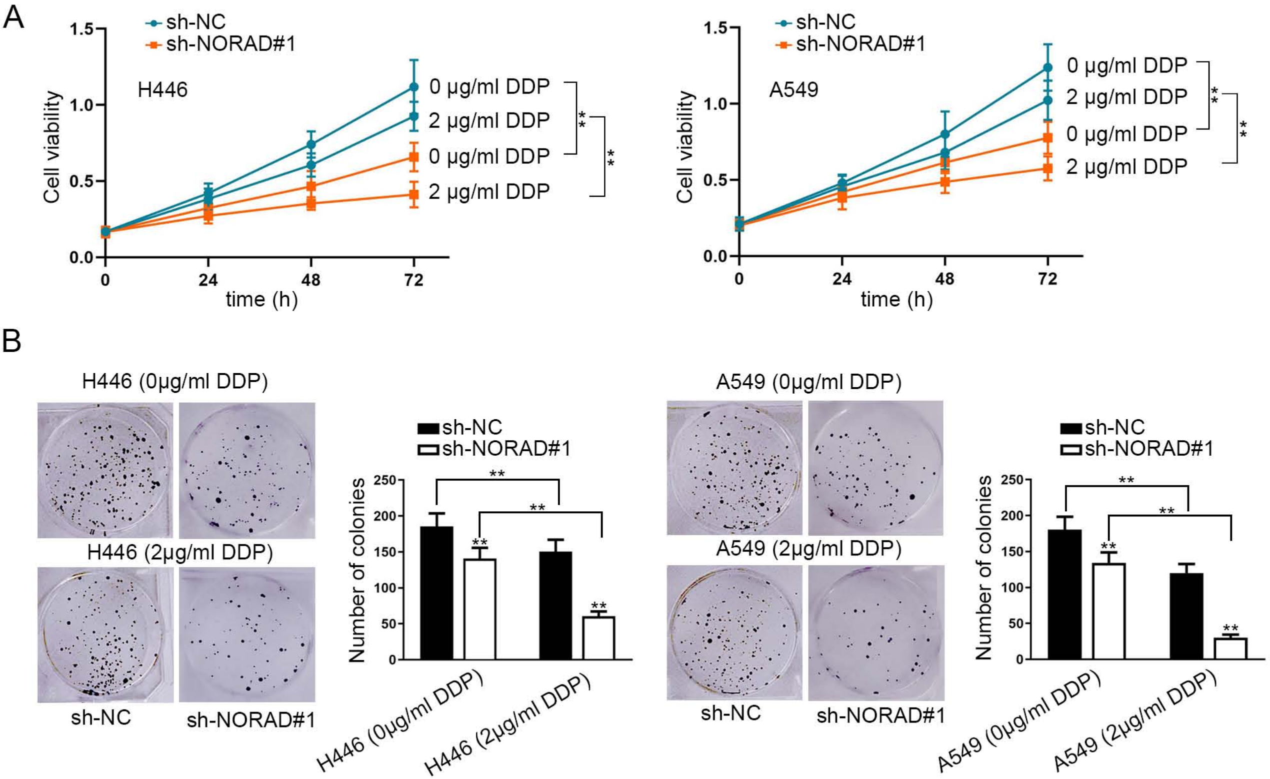
A
sh-NC
sh-NORAD#1
H446
Cell viability
0 μg/ml DDP
2 μg/ml DDP
0 μg/ml DDP
2 μg/ml DDP
**
**
time (h)
A549
B
H446 (0μg/ml DDP)
H446 (2μg/ml DDP)
sh-NC
sh-NORAD#1
Number of colonies
H446 (0μg/ml DDP)
H446 (2μg/ml DDP)
A549 (0μg/ml DDP)
A549 (2μg/ml DDP)
A549 (0μg/ml DDP)
A549 (2μg/ml DDP)

Supplementary figure1

A. MTT assays were conducted to evaluate cell viability in H449 and A549 cell lineswith or without DDP treatment. B. Colony formation assays were performed to assess cell proliferative ability with or without DDP treatment in H449 and A549 cell lineswith or without DDP treatment. **P< 0.01.
